# Pembrolizumab and trastuzumab in combination with FLOT in the perioperative treatment of HER2-positive, localized esophagogastric adenocarcinoma—a phase II trial of the AIO study group (AIO STO 0321)

**DOI:** 10.3389/fonc.2023.1272175

**Published:** 2023-10-16

**Authors:** Joseph Tintelnot, Alexander Stein, Salah-Eddin Al-Batran, Thomas Ettrich, Thorsten Götze, Barbara Grün, Georg Martin Haag, Vera Heuer, Ralf-Dieter Hofheinz, Nils Homann, Tobias Sebastian Bröring, Mariana Santos Cruz, Annika Kurreck, Sylvie Lorenzen, Nicolas Moosmann, Christian Müller, Markus Schuler, Gabriele Siegler, Mascha Binder, Eray Gökkurt

**Affiliations:** ^1^ ll. Department of Medicine, University Medical Center Hamburg-Eppendorf, Hamburg, Germany; ^2^ Hematology-Oncology Practice Eppendorf (HOPE), Hamburg, Germany; ^3^ Institute of Clinical Cancer Research, Krankenhaus Nordwest, Frankfurt, Germany; ^4^ l. Department of Medicine, University Hospital Ulm, Ulm, Germany; ^5^ Department of Medical Oncology, University Medical Center Heidelberg, Heidelberg, Germany; ^6^ Department of Oncology, St. Anna Hospital Herne, Herne, Germany; ^7^ Medical Department, University Medical Center Mannheim, Mannheim, Germany; ^8^ ll. Medical Department, Klinikum Wolfsburg, Wolfsburg, Germany; ^9^ Charité – Universitätsmedizin Berlin, corporate member of Freie Universität Berlin and Humboldt-Universität zu Berlin, Department of Hematology, Oncology, and Cancer Immunology, Berlin, Germany; ^10^ Rechts der Isar Hospital, Technical University of Munich, Munich, Germany; ^11^ Department of Oncology and Hematology, Barmherzige Brüder Regensburg Hospital, Regensburg, Germany; ^12^ Department of Hematology and Oncology, Klinik Essen-Mitte, Essen, Germany; ^13^ Onkologischer Schwerpunkt am Oskar-Helene-Heim, Berlin, Germany; ^14^ Department of Internal Medicine V, Hematology/Oncology, Hospital Nürnberg Nord/Paracelsus Medical University, Nürnberg, Germany; ^15^ Division of Medical Oncology, University Hospital Basel, Basel, Switzerland

**Keywords:** esophagogastric adenocarcinoma, HER2, trastuzumab, pembrolizumab, perioperative

## Abstract

**Background:**

Esophagogastric adenocarcinoma (EGA) presents a substantial global health challenge as the number of cases continues to rise. The current standard approach for treating localized EGA involves a combination of triplet chemotherapy, which consists of a platinum compound, a fluoropyrimidine, and a taxane (known as FLOT), followed by surgery. In cases of metastatic EGA with HER2-positive status or in certain studies with localized EGA, the use of HER2-targeted antibodies such as trastuzumab has shown improved responses. Recently, the addition of programmed cell death protein 1 (PD-1) inhibitors, such as pembrolizumab, when combined with 5-FU, platinum-based chemotherapy, and trastuzumab, has demonstrated significant enhancements in response rates for HER2-positive metastatic EGA. However, there is currently insufficient evidence regarding this treatment approach in localized HER2-positive disease.

**Methods:**

The PHERFLOT study is an open-label, single-arm, multicenter, exploratory phase II trial designed to assess the efficacy, safety, and tolerability of perioperative pembrolizumab, FLOT, and trastuzumab in patients with previously untreated localized HER2-positive EGA. In total, 30 patients will be recruited. The co-primary end points are pathological complete response rate and disease-free survival rate after 2 years. Secondary objectives include safety and tolerability, efficacy in terms of progression-free survival and objective response rate and translational markers, such as blood-based signatures (e.g., immune repertoire changes or emergence of anti-HER2 resistance variants) or microbiota signatures that may correlate with immune activation and therapy response.

**Discussion:**

Recent evidence from phase II clinical trials demonstrated improved efficacy through the addition of trastuzumab to perioperative FLOT. Furthermore, in advanced or metastatic EGA, the combination of trastuzumab, FLOT, and the PD1-inhibitor pembrolizumab significantly improved treatment response. The PHERFLOT study aims to assess the efficacy and safety of this treatment approach in HER2-positive–localized EGA, potentially identifying a promising new perioperative regimen for localized EGA, which then needs to be confirmed within a randomized trial. Furthermore, the accompanying translational program of the study might help to improve the stratification of suitable patients and to identify potential translational targets for future clinical trials.

**Clinical trial registration:**

https://clinicaltrials.gov, identifier NCT05504720.

## 1 Introduction

With more than 1.5 million cases in 2018, esophageal and gastric cancers belong to the most common malignancies worldwide. Both diseases are associated with a high disease-related mortality, resulting in approximately 1.3 million deaths per year (1). FLOT, a triplet chemotherapy consisting of 5-fluorouracil (5-FU), leucovorin, oxaliplatin, and docetaxel, represents one of the most intensively investigated regimens for gastric and gastroesophageal junction (GEJ) cancer. It has been evaluated in the metastatic setting, in the limited metastatic setting (2), in elderly patients, and in primarily operable patients (2–5). The AIO FLOT4 phase II/III study evaluated FLOT versus Epirubicin, Cisplatin, and 5-FU (ECF)/Epirubicin, Cisplatin, and Capecitabine (ECX) in 716 patients with resectable gastric or GEJ cancer. The phase II part of the randomized phase II/III FLOT4 trial demonstrated a significantly improved histopathological regression grade in patients receiving FLOT compared to ECF (6% vs. 16%). Additionally, the rate of pathological complete response (pCR) or subtotal regression (TRG1a/b) was significantly increased with FLOT 37% versus ECF 23% (5). The phase III part of this trial demonstrated an improved overall survival (OS) in the FLOT group compared to the ECF/EXC group (median OS 50 months vs. 35 months). In summary, FLOT is considered the standard chemotherapy regimen for gastric cancer in the perioperative setting and is, thus, serving as the chemotherapeutic backbone in the underlying clinical trial.

### 1.1 Immunotherapy in gastric cancers

During the past years, several first-line phase III trials confirmed the safety and efficacy of PD-1 or programmed death-ligand 1 (PD-L1) inhibition in esophagogastric adenocarcinoma (EGA). Pembrolizumab alone or in combination with chemotherapy was examined among patients with PD-L1 combined positive score (CPS) (combined prognostic score) ≥ 1 within the KEYNOTE-062 trial. In this study, pembrolizumab demonstrated non-inferiority in comparison to chemotherapy alone in terms of OS. However, the objective response rate (ORR) and the progression-free survival (PFS) were numerically inferior to chemotherapy alone (6). However, among patients with a PD-L1 CPS ≥ 10 an improvement in OS compared to chemotherapy was observed (exploratory analysis). The combination of chemotherapy and pembrolizumab showed a trend toward improved efficacy without a statistically significant improvement in OS and PFS (6). The CheckMate 649 trial investigated first-line nivolumab plus chemotherapy versus chemotherapy alone. The addition of nivolumab to FOLFOX chemotherapy resulted in an OS benefit for patients with a PD-L1 CPS expression ≥ 5 (primary end point) (7). Furthermore, the combination of pembrolizumab and cisplatin-/fluoropyrimidine-based chemotherapy investigated within the KEYNOTE-590 trial in patients with squamous cell esophageal cancer (73%) as well as adenocarcinomas of the esophagus and GEJ (Siewert Type 1) demonstrated superior OS compared to chemotherapy alone among patients with a PD-L1 CPS expression ≥10. Similarly, the positive effect of chemotherapy and PD-L1 combination was confirmed by the results of the KEYNOTE-859 trial including EGA only. The significant survival benefit of adding immunotherapy to standard chemotherapy was only observed in EGA patients with a CPS ≥10 and CPS ≥5, respectively (8). In summary, these findings led to the approval of PD-1 inhibitors in combination with 5-FU and platinum chemotherapy in EGA with a CPS ≥5 for nivolumab and a CPS ≥10 for pembrolizumab in esophageal and HER2-AC GEJ by the European Medicines Agency (EMA) or without CPS restriction by the Food and Drug Administration (FDA). In the perioperative context, ongoing phase III trials, MATTERHORN, and KEYNOTE-585 are presently investigating the incorporation of durvalumab or pembrolizumab alongside chemotherapy (9, 10). Preliminary information from respective press releases suggests a potential enhancement in pathological response rates.

### 1.2 HER2-targeting in EGA

The presence of HER2 overexpression assumes a pivotal role in the prognosis and predictive assessment of outcomes in EGA. It is identified by either 3+ staining or 2+ staining coupled with positive *in-situ* hybridization (ISH) results. Approximately 10%–30% of all gastric or EGA cases manifest HER2-protein overexpression or HER2-gene amplification (11, 12). It is noteworthy that HER2 overexpression is more commonly observed in gastroesophageal junction (GEJ) cancer than in gastric cancer, particularly in cases classified as intestinal type according to the Lauren classification (11, 12). The ToGa study investigated trastuzumab in combination with chemotherapy versus chemotherapy alone for the treatment of HER2-positive advanced gastric or GEJ cancer demonstrating an improved OS for the combination therapy (13). These findings led to the approval of chemotherapy plus trastuzumab in the treatment of HER2-positive advanced stomach cancer by the FDA and EMA. In the perioperative treatment of HER2-positive locally advanced EGA, the combination of trastuzumab and FLOT was found to be safe and active resulting in more than 20% complete pathological responses (14). Furthermore, the addition of trastuzumab and pertuzumab, another HER2 antibody, to perioperative FLOT significantly improved the pCR and rates of nodal negativity in HER2-positive, primarily resectable EGA (PETRARCA trial) (15). Moreover, another phase ll trial showed improved major pathological response rates through the addition of trastuzumab to perioperative chemotherapy while showing no added value for the simultaneous addition of pertuzumab (INNOVATIVE trial) (16). Furthermore, in advanced gastric cancer, the use of HER2-targeting antibodies with drug conjugates has recently emerged as a new standard of treatment in later treatment lines (17, 18).

### 1.3 Increasing anti-tumor immune reactions by chemotherapy and HER2-targeting

The HER2-receptor antibody trastuzumab induces both antibody-dependent cytotoxicity and lymphoid infiltration into the tumor tissue (19). Recent preclinical data demonstrated the synergistic effect of combining HER2 blockade with immune checkpoint inhibition, that is, HER2 blockade by the antibody-drug conjugate trastuzumab-emtansine, leading to an improved PD-1 and CTLA4 blockade in an orthotopic breast cancer model (20). Moreover, in metastatic patients, the combination of CAPOX, trastuzumab, and pembrolizumab resulted in an ORR of 91% in HER2-positive disease (21). Recently published data of the PANTHERA and INTEGA trials confirmed the high efficacy of combining trastuzumab, pembrolizumab, and chemotherapy already reported from two other single-arm phase II trials (22, 23). Furthermore, the phase III KEYNOTE-811 trial demonstrated a significantly increased ORR through the addition of pembrolizumab to chemotherapy and trastuzumab (51.9%–74.4%) (24). This advantage was particularly pronounced among patients with tumors exhibiting a CPS score of ≥1, ultimately resulting in the FDA- and EMA-granting approval for this combination as a first-line therapy.

### 1.4 Translational background and work-up

Predictive markers to tailor treatment are urgently warranted either at baseline or early during antitumor treatment. First, we will evaluate strategies to predict the outcome of checkpoint inhibition by liquid biopsy immunoprofiling at baseline and shortly after the initiation of study treatment further correlating this with tumor regression and OS. Tumor-infiltrating lymphocyte (TiL) repertoires will be determined by next-generation sequencing (NGS) of the T-cell receptor beta (TCRβ) and immunoglobulin heavy locus (IGH). In previous studies, the diversification of T cells during therapy or TiL clone expansion in the peripheral blood was associated with response to immunotherapies (25, 26). Resistance to HER2-targeting in HER2-positive tumors might be already present at time of first diagnosis or will eventually develop during antitumor treatment. Several mechanisms of treatment resistance have already been discussed in current literature, particularly the loss of HER2 amplification (27). Therefore, baseline formalin-fixed, paraffin embedded (FFPE) and circulating tumor DNA (ctDNA) will be assessed for HER2 overexpression (immunohistochemistry [IHC] and ISH in FFPE) and HER-signaling alterations (amplifications and/or mutations in, e.g., EGFR, HER2, HER3, and PIK3CA). The following genes are covered by the translational analysis: CD274 (PD-L1), TP53, ERBB2, JAK1, JAK2, B2M, ARID1A, CTNNB1, ERBB4, FCGR2A, FCGR3A, KRAS, SMAD4, PIK3CA, APC, FAT4, KMT2C, and LRP1B.

The gut microbiota consists of trillions of bacteria and was recently introduced as a key factor for response to checkpoint inhibitors in melanoma and lung cancer as well as chemotherapeutic treatment in colorectal cancer (28–30). These microbes can either directly control an anti-tumor immune response within the intestine or interact with immune and tumor cells within the tumors. We will therefore analyze the oral and intestinal microbiome by shotgun metagenomic sequencing and/or 16S rRNA sequencing prior to treatment initiation, at time of surgery, and following the completion of chemotherapy to explore the stability of the microbiota during treatment, to compare the different microbiota to the intra-tumoral microbiota at time of surgery (from FFPE tissue) and to correlate bacterial species and diversity patterns with response to therapy potentially identifying innovative biomarkers.

## 2 Main

### 2.1 Study objective

The two co-primary objectives of this phase II study will be to demonstrate an improvement in the pCR rate compared to historical controls (interim read out after surgery of last patient in study with 18 months recruitment after 24 months) and in disease-free survival (DFS) according to RECIST v1.1. Secondary objectives consist of additional efficacy and tolerability parameters, namely, ORR according to RECIST v1.1, DFS according to RECIST v1.1, R0 resection rate, OS, safety according to CTCAE, tolerability (including perioperative morbidity), and translational markers.

### 2.2 Methods/design

The PHERFLOT study is an open-label, single-arm, multicenter, and exploratory phase II trial designed to assess efficacy, safety, and tolerability of pembrolizumab, trastuzumab, and FLOT as perioperative treatment of HER2-positive–localized EGA. Thirty patients will be recruited over a period of 18 months. Follow-up regarding survival parameters will be performed for a maximum of 24 months from inclusion of the last patient. Participating hospitals are located in Germany.

### 2.3 Treatment

**Figure 1:**
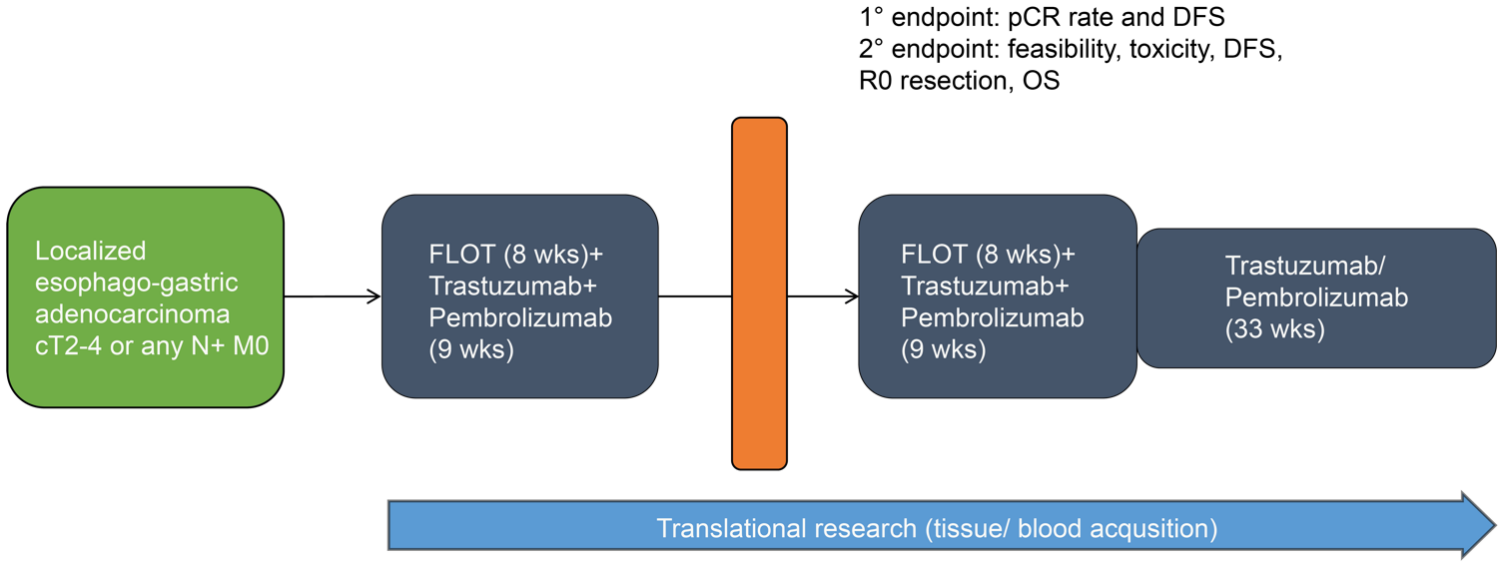
Study schedule overview.

All eligible patients will receive pembrolizumab at an absolute dosage of 200 mg in combination with trastuzumab (6 mg/kg following a loading dose of 8 mg/kg) every 3 weeks and 5-FU 2600 mg/m^2^ for 24h, folinic acid 200 mg/m^2^, oxaliplatin 85 mg/m^2^, and docetaxel 50 mg/m^2^ (FLOT regimen) every 2 weeks for 8 weeks. This will be followed by surgical resection 4 weeks after the last preoperative treatment at the earliest. Within 4–10 weeks following surgery, another 8 weeks of perioperative combination treatment will be administered. Thereafter, pembrolizumab 200 mg and trastuzumab 6 mg/kg will be given alone for up to 11 cycles. This totals up to 1 year of systemic treatment (maximum of 17 cycles of pembrolizumab/trastuzumab per patient including pre- and postoperative chemo-immunotherapy, **Figure 1**).

**Figure 2:**
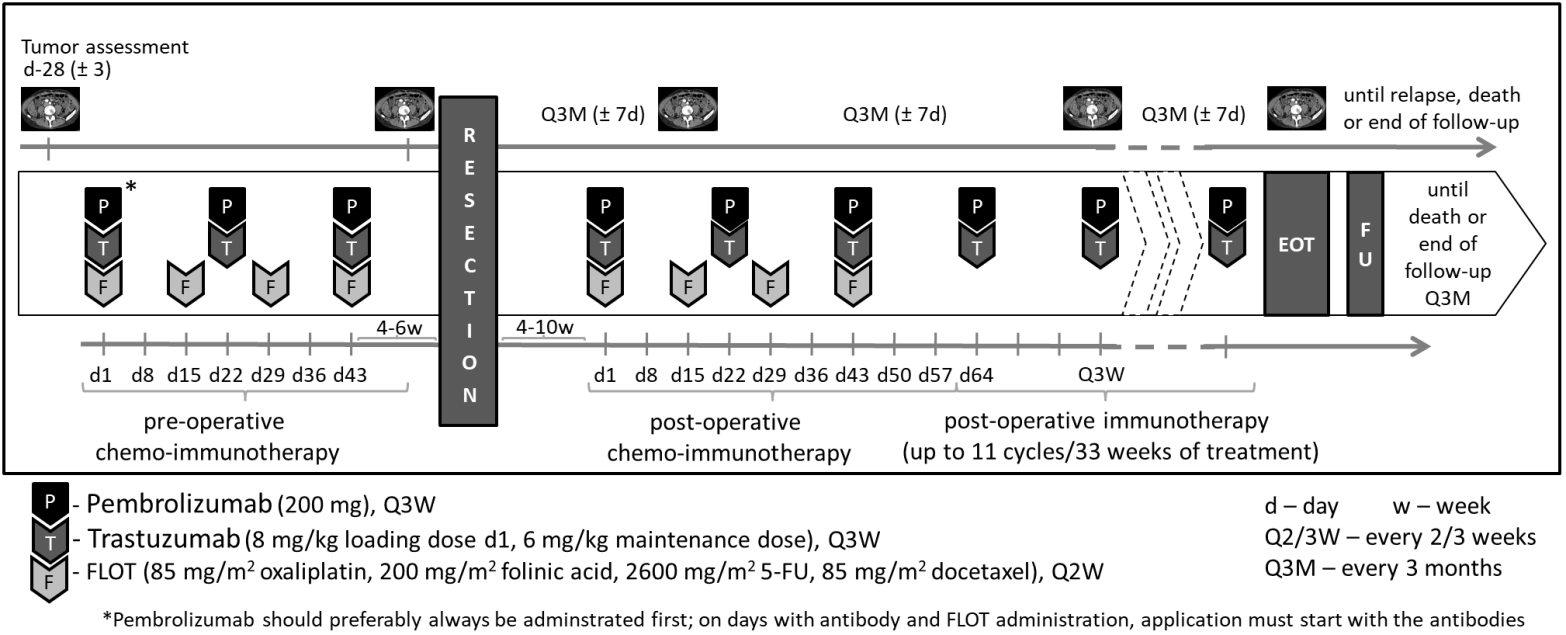
Treatment overview.

Treatment will continue until disease relapse/progressive disease (PD), unacceptable adverse events (AEs), intercurrent illness that prevents from further administration of treatment, investigator’s decision to withdraw the subject, patient’s wish or withdrawal, pregnancy of the subject, non-compliance with study intervention or procedure requirements, administrative reasons requiring termination of treatment, or completion of treatment per protocol. The intervention(s) to be used in this trial is/are outlined below in **Table 1**. The overall treatment schedule is summarized in **Figure 2**.

**Table 1:** Trial interventions.

Drug	Dose/potency	Duration of administration	Route of administration	Day(s) of application*
Perioperative chemo-immunotherapy phase (8 weeks pre- and 8 weeks post-surgery):				
**Pembrolizumab** **Trastuzumab** **FLOT:** ** Oxaliplatin** Folinic Acid** 5-FU** ** Docetaxel**	200 mg 8 mg/kg (loading dose) 6 mg/kg 85 mg/m² 200 mg/m² 2600 mg/m² * 85 mg/m²	30 min 90 min 30 min 2 h 1 h 24 h 1 h	IV infusion IV infusion IV infusion IV infusion IV infusion IV infusion IV infusion	d1, d22, d43 d1 d22, d43 d1, d15, d29, d43
Post-chemotherapy phase (for up to 33 weeks/11 cycles):				
**Pembrolizumab** **Trastuzumab**	200 mg 6 mg/kg	30 min 30 min	IV infusion IV infusion	d1 of each 3-week cycle d1 of each 3-week cycle

*Therapy can also be administered over 2 days with pembrolizumab/trastuzumab on first day and FLOT on following day at time points where combination is planned. Infusion rates of chemotherapeutical components might be modified according to local standards.

**Folinic acid can be administered according to local standards (product and dosing).

***Dosage in dihydropyrimidine dehydrogenase (DPD) mutation carriers with a CPIC activity score of 1.0–1.5 should be reduced by 50%.

Participants are eligible to be included in the study only if all of the following criteria apply:

1. The participant provides written informed consent for the trial.

2. Male/female* participants who are at least 18 years of age on the day of signing informed consent.

ο *There are no data that indicate special gender distribution. Therefore, patients will be enrolled in the study gender independently.

3. In the investigator’s judgement, participant is willing and able to comply with the study protocol including the planned surgical treatment.

4. Histologically confirmed adenocarcinoma of the GEJ (Type I-III according to Sievert’s classification) or the stomach (cT2, cT3, cT4, any N category, M0), or (any T, N+, M0) that:

• is not infiltrating any adjacent organs or structures by computed tomography (CT or MRI) evaluation.

• does not involve peritoneal carcinomatosis.

• is considered medically and technically resectable.

ο Note: the absence of distant metastases must be confirmed by CT or MRI of the thorax and abdomen and, if there is clinical suspicion of osseous lesions, a bone will be scanned. If peritoneal carcinomatosis is suspected clinically, its absence must be confirmed by laparoscopy. Diagnostic laparoscopy is mandatory in patients with T3 or T4 tumors of the diffuse type histology in the stomach.

5. Participants must have HER2-positive disease defined as either IHC 3+ or IHC 2+, the latter in combination with ISH+, as assessed locally on primary tumor.

6. Participants must be candidates for potential curative resection as determined by the treating surgeon.

7. No prior systemic anti-cancer therapy (e.g., cytotoxic or targeted agents or radiotherapy).

8. No prior partial or complete esophagogastric tumor resection.

9. ECOG (Eastern Cooperative Oncology Group) performance status score of 0 or 1.

10. Male participants: A male participant must agree to use a contraception during the treatment period and for at least 6 months after the last dose of study intervention and refrain from donating sperm during this period.

ο Female participants: A female participant is eligible to participate if she is not pregnant, not breastfeeding, and at least one of the following conditions applies:

- Not a woman of childbearing potential (WOCBP).

ο OR

- A WOCBP who agrees to follow the contraceptive guidance during the treatment period and for at least 7 months after the last dose of study intervention.

11. Participants have adequate organ function as defined in the following table (**Table 2**). Specimens must be collected within 14 days prior to enrolment (also to be repeated if older than 14 days at day of first treatment).

**Table 2:** Adequate organ function laboratory values.

System	Laboratory value
Hematological	
Absolute neutrophil count (ANC)	≥ 1500/µL
leucocytes	≥ 3000/µL
Thrombocytes	≥ 100000/µL
Hemoglobin	≥ 9.0 g/dL or ≥ 5.6 mmol/L^a^
Renal: Measured or calculated^b^ creatinine clearance (CrCl)	≥ 50 mL/min
Hepatic	
Total bilirubin	≤ 1.5 × ULN OR direct bilirubin ≤ ULN for participants with total bilirubin levels > 1.5 × ULN
AST (SGOT) and ALT (SGPT)	≤ 2.5 × ULN
Coagulation	
International normalized ratio (INR) OR prothrombin time (PT) and activated partial thromboplastin time (aPTT)	≤ 1.5 × ULN unless participant is receiving anticoagulant therapy as long as PT or aPTT is within therapeutic range of intended use of anticoagulants

ALT (SGPT), alanine aminotransferase (serum glutamic pyruvic transaminase); AST (SGOT), aspartate aminotransferase (serum glutamic oxaloacetic transaminase); GFR, glomerular filtration rate; ULN, upper limit of normal. ^a^Criteria must be met without erythropoietin dependency and without packed red blood cell (pRBC) transfusion within the past 2 weeks. ^b^CrCl should be calculated per institutional standard.

Participants are excluded from the study if any of the following criteria apply:

1. Participants with involved retroperitoneal (e.g., para-aortal, paracaval, or interaortocaval lymph nodes) or mesenterial lymph nodes (distant metastasis)!
2. A WOCBP who has a positive urine pregnancy test within 72h prior to start of study intervention. If the urine test is positive or cannot be confirmed as negative, a serum pregnancy test will be required.
3. Received prior therapy with an anti–PD-1, anti–PD-L1, or anti–PD-L2 agent or with an agent directed to another stimulatory or co-inhibitory T-cell receptor (e.g., CTLA-4, OX-40, and CD137).
4. Participant received colony-stimulating factors [e.g., granulocyte colony-stimulating factor (G-CSF), granulocyte-macrophage colony-stimulating factor (GM-CSF) or recombinant erythropoietin] within 28 days prior to the first dose of study intervention.
5. Major surgery within 2 weeks of starting study intervention and patients must have recovered from any effects of any major surgery.
6. Concomitant use of drugs inhibiting (dihydropyrimidine dehydrogenase) DPD activity (including sorivudine and brivudine), the required wash out phase is 4 weeks before start of the study intervention.
7. Inadequate cardiac function (LVEF value <55%) as determined by echocardiography.
8. Resting ECG indicating uncontrolled, potentially reversible cardiac conditions, as judged by the investigator (e.g., unstable ischemia, uncontrolled symptomatic arrhythmia, congestive heart failure, QTcF prolongation >500 ms, and electrolyte disturbances), or patients with congenital long QT syndrome.
9. Participant has received a live vaccine or live-attenuated vaccine within 30 days prior to the first dose of study drug. Administration of killed vaccines is allowed.
10. Participant is currently participating in or has participated in a study of an investigational agent or has used an investigational device within 4 weeks prior to the first dose of study intervention.
11. Participant has a diagnosis of immunodeficiency or is receiving chronic systemic steroid therapy (in dosing exceeding 10 mg daily of prednisone equivalent) or any other form of immunosuppressive therapy within 7 days prior to the first dose of study drug.
12. Participant has a known additional malignancy that is progressing or has required active treatment within the past 2 years. Participants with basal cell carcinoma of the skin, squamous cell carcinoma of the skin or carcinoma *in situ* (e.g., breast carcinoma and cervical cancer *in situ*) that have undergone potentially curative therapy are not excluded.
13. Participant has myelodysplastic syndrome (MDS)/acute myeloid leukemia (AML) or with features suggestive of MDS/AML.
14. History of severe allergic, anaphylactic, or other hypersensitivity reactions to chimeric or humanized antibodies or fusion protein; known hypersensitivity to Chinese hamster ovary cell products or to any component of the pembrolizumab or trastuzumab formulation.
15. Any known contraindication (including hypersensitivity) to docetaxel, 5-FU, folinic acid/leucovorin, or oxaliplatin.
16. Known DPD deficiency. Patients with a reduced DPD activity (CPIC activity score of 1.0–1.5) might participate in the study and receive a reduced dosage of 5-FU after discussion with the coordinating investigator and sponsor (https://cpicpgx.org/guidelines/guideline-for-fluoropyrimidines-and-dpyd/).
17. Participant has active autoimmune disease that has required systemic treatment in the past 2 years (i.e., with the use of disease-modifying agents, corticosteroids, or immunosuppressive drugs). Replacement therapy (e.g., thyroxine, insulin, or physiologic corticosteroid replacement therapy for adrenal or pituitary insufficiency) is not considered a form of systemic treatment and is allowed.
18. Participant has a history of (non-infectious) pneumonitis/interstitial lung disease that required steroids or has current pneumonitis/interstitial lung disease.
19. Participant has an active infection requiring systemic therapy.
20. Participant has a known history of human immunodeficiency virus (HIV) infection.
21. Participant has a known history of Hepatitis B [defined as Hepatitis B surface antigen (HBsAg) reactive] or known active Hepatitis C virus (defined as HCV RNA is detected) infection.
22. Participant is considered a poor medical risk due to a serious, uncontrolled medical disorder, non-malignant systemic disease or active, uncontrolled infection. Examples include, but are not limited to, uncontrolled ventricular arrhythmia, recent (within 3 months) myocardial infarction, uncontrolled major seizure disorder, unstable spinal cord compression, superior vena cava syndrome, extensive interstitial bilateral lung disease on high-resolution computed tomography (HRCT) scan, previous allogenic bone marrow/blood transplantation or any psychiatric disorder or substance abuse that prohibits obtaining informed consent.
23. Participant is pregnant or breastfeeding or expecting to conceive or father children within the projected duration of the study, starting with the screening visit through 6 months after the last dose of study intervention.
24. Participant has had an allogenic tissue/solid organ transplant.

### 2.4 Safety monitoring

There will be a near real-time monitoring of safety parameters (e.g., SAEs reported) by a continuous toxicity monitoring board for the first six patients enrolled to immediately identify any risks for patient safety. In addition to these *ad-hoc* scheduled meetings depending on SAE reporting, the toxicity monitoring board will meet after the first six patients have finished the third treatment of pembrolizumab and trastuzumab plus FLOT and have passed their presurgical assessment to re-evaluate the risk-benefit-ratio of the study and to provide a recommendation on the continuation of the study to the coordinating investigator and the sponsor. Recruitment can be halted at the discretion of the toxicity monitoring board.

#### 2.4.1 Adverse event monitoring

The investigator or qualified designee will assess each participant to evaluate for potential new or worsening AEs as specified in the schedule of activities and more frequently if clinically indicated. AEs will be graded and recorded throughout the study and during the follow-up period according to NCI CTCAE v5.0. Toxicities will be characterized in terms of seriousness, causality, toxicity grading, and action taken with regard to study intervention.

#### 2.4.2 Examination

##### 2.4.2.1 Full physical exam

The investigator or qualified designee will perform a complete physical exam during the screening period within 14 days prior to first study drug administration. Clinically significant abnormal findings should be recorded as medical history. Additional full physical exams should be performed, that is, before and after surgery and at EOT. Height will be measured at screening only. After the first dose of study intervention, new clinically significant abnormal findings should be recorded as AEs. Investigators should pay attention to clinical signs related to previous serious illnesses.

##### 2.4.2.2 Directed physical exam

For cycles that do not require a full physical exam as per the schedule of activities, the investigator or qualified designee will perform a directed physical exam as clinically indicated prior to study intervention administration. After the first dose of study intervention, new clinically significant abnormal finding should be recorded as AEs. Investigators should pay attention to clinical signs related to previous serious illnesses.

##### 2.4.2.3 Vital signs

The investigator or qualified designee will take vital signs at screening, prior to the administration of each dose of study intervention and at treatment discontinuation. Vital signs should include temperature, pulse, respiratory rate, blood pressure and oxygen saturation. Since infusion-related reactions are known potential side effects of pembrolizumab/trastuzumab, the patients must be closely monitored during and after infusion as described below:

##### 2.4.2.4 12-Lead electrocardiogram

A standard 12-lead electrocardiogram (ECG) will be performed at screening, before surgery, at EOT and whenever clinically indicated using local standard procedures. Clinically significant abnormal findings from screening visit should be recorded as medical history, clinically significant abnormal findings during treatment should be recorded as AE.

##### 2.4.2.5 Echocardiography

Echocardiography will be performed at screening, after surgery/before start of post-operative treatment, at start of pembrolizumab/trastuzumab only treatment and then every 3 months during trastuzumab treatment. If treatment with trastuzumab is permanently discontinued, no further echocardiography is necessary.

##### 2.4.2.6 Eastern Cooperative Oncology Group performance scale

The investigator or qualified designee will assess ECOG status at screening, prior to the administration of each dose of study intervention and discontinuation of study intervention.

### 2.5 Imaging

#### 2.5.1 Initial tumor imaging

Initial tumor imaging at screening must be performed within 28 days ( ± 3 days) prior to start of study intervention. The site study team must review screening images to confirm the participant has measurable or evaluable disease per RECIST 1.1.

Tumor imaging performed as part of routine clinical management is acceptable for use as screening tumor imaging if they are suitable for baseline RECIST data collection and performed within 28 days prior to the start of study intervention.

#### 2.5.2 Tumor imaging during the study

Tumor imaging is strongly preferred to be acquired by CT. For the abdomen, contrast-enhanced magnetic resonance imaging (MRI) may be used when CT with iodinated contrast is contraindicated, or when local practice mandates it and if clinically appropriate. MRI is the strongly preferred modality for imaging the brain. A change between the imaging techniques is allowed as long as an assessment of the objective response or recurrency is possible. Imaging should include the chest and abdomen at baseline and all subsequent imaging time points.

The first on-study imaging assessment should be performed between the completion of the last pre-operative FLOT chemotherapy cycle/immunotherapy and surgery. Subsequent tumor imaging should be performed every 3 months ( ± 7 days) or more frequently if clinically indicated. After 2 years (from EOT), imaging may be performed every 6 months.

Imaging timing should follow calendar days and should not be adjusted for delays in treatment cycle starts. Imaging should continue to be performed until disease progression/relapse is identified by the investigator.

#### 2.5.3 End of treatment and follow-up tumor imaging

Subjects who discontinue study treatment for reasons other than tumor relapse/PD will have post-treatment follow-up for disease status until tumor relapse/PD, initiating a non-study cancer treatment, withdrawing consent, or becoming lost to follow up. All subjects will be followed for OS until death, withdrawal of consent, loss to follow up, or the end of the study.

After the last administration of study medication, each subject will be followed for 30 days for AE monitoring. Serious adverse events (SAEs) and events of clinical interest (ECIs) will be collected for 90 days after the last administration of study medication or for a minimum of 30 days after the end of treatment if the subject has initiated a new anticancer therapy, whichever is earlier.

In participants who discontinue study treatment, tumor imaging should be performed at the time of treatment discontinuation ( ± 4 days window). If previous imaging was obtained within 4 weeks prior to the date of discontinuation, then imaging at treatment discontinuation is not mandatory. In participants who discontinue study treatment due to documented disease progression/relapse, this is the final required tumor imaging.

For participants who discontinue study treatment without documented disease progression/relapse confirmed by RECIST 1.1, tumor imaging should be performed at the time of treatment discontinuation ( ± 4 weeks). Then every effort should be made to continue monitoring their disease status by tumor imaging (every 3 months ±7 days; after 2 years, every 6 months ±7 days) to monitor disease status until the start of a new anticancer treatment, disease progression/relapse, pregnancy, death, withdrawal of consent, or the end of the study, whichever occurs first.

### 2.6 Visits

#### 2.6.1 Safety follow-up visit

The mandatory safety follow-up visit should be conducted approximately 30 days ( ± 7 days) after discontinuation or before the initiation of a new anti-cancer treatment, whichever comes first. All AEs that occur prior to the safety follow-up visit should be recorded. Participants with an AE of Grade >1 will be followed until the resolution of the AE to grades 0–1 or until the beginning of a new anti-cancer therapy, whichever occurs first. SAEs that become known to the trial site within 90 days of the end of treatment or before initiation of a new anti-cancer treatment (whichever occurs first) should also be followed and recorded. The following procedures must be performed during the safety follow-up visit:

- Review/documentation of prior and concomitant medication.
- Review AEs.
- Post-study anti-cancer therapy status.
- Vital signs (should include temperature, pulse, respiratory rate, and blood pressure).
- ECOG performance status.
- WOCBP: pregnancy test—urine or serum β-HCG; to be performed monthly and when expected menstrual cycle is missed or when pregnancy is otherwise suspected until the end of relevant systemic exposure to the study drug (i.e., up to 7 months after last trastuzumab dose) in accordance with the CTFG guidance on contraception.
- CBC with differential: White blood cell (WBC) count with differential and absolute neutrophil count (ANC); absolute lymphocyte count (ALC); red blood cells (RBCs); platelet count; hemoglobin; hematocrit.
- Comprehensive serum chemistry panel: albumin; alkaline phosphatase; alanine aminotransferase (ALT); aspartate aminotransferase (AST); lactate dehydrogenase (LDH); uric acid; calcium; glucose; phosphorus; potassium; sodium; magnesium; total bilirubin; direct bilirubin (if total bilirubin is elevated above the upper limit of normal); total protein; blood urea nitrogen; C-reactive protein (CRP); gamma-GT; creatinine, lipase.

#### 2.6.2 Efficacy follow-up visits

Participants who complete the protocol-required study intervention or who discontinue study intervention for a reason other than tumor relapse/PD will begin the efficacy follow-up phase and should be assessed every 3 months ( ± 7 days) by radiologic imaging to monitor disease status. After 2 years since EOT, imaging will be done every 6 months ( ± 7 days). Every effort should be made to collect information regarding disease status until the start of a new anti-cancer therapy, tumor relapse/PD, death, or end of the study. Information regarding post-study anti-cancer treatment will be collected if new treatment is initiated. The following procedures must be performed during the follow-up visit:

- Post-study anti-cancer therapy status.
- WOCBP: pregnancy test—urine or serum β-HCG; to be performed monthly and when expected menstrual cycle is missed or when pregnancy is otherwise suspected until the end of relevant systemic exposure to the study drug (i.e., up to 7 months after last trastuzumab dose), in accordance with the CTFG guidance on contraception.

#### 2.6.3 Survival follow-up

Participants who experience confirmed tumor relapse/PD or start a new anticancer therapy, will move into the survival follow-up phase and should be contacted within regular visits or by telephone every 3 months ( ± 7 days) to assess for post-study anti-cancer therapy status and survival status until death, withdrawal of consent, or the end of the study, whichever occurs first.

### 2.7 Translational analysis

#### 2.7.1 Tumor tissue

Archival tumor tissue sample or newly (within clinical routine) obtained core or excisional biopsy of a tumor lesion not previously irradiated should be sent to a central pathology laboratory for optional accompanying research project (baseline sample). FFPE tissue blocks are preferred over tumor slides. Newly obtained biopsies are preferred over archived tissue. Core or excisional biopsies are mandatory (fine needle aspiration and bone metastasis samples are not acceptable). Within the accompanying research project (Section 8 of the study original study protocol) PD-L1 CPS will be determined through a standardized assay (22C3 pharmDx assay, DAKO North America) by the central pathology. The primary and all meaningful secondary end points will be analyzed with a subpopulation presenting with PD-L1 CPS ≥1, if differing from the total population.

#### 2.7.2 Translational research blood samples

Blood samples will be taken prior to treatment, prior to second pembrolizumab administration, pre- as well as postoperatively, and afterwards every 3 months until tumor relapse/PD.

#### 2.7.3 Translational research stool and saliva samples

Stool and saliva samples will be collected prior to treatment, preoperatively, directly after the completion of postoperative chemotherapy, and 3 months after completing postoperative treatment.

### 2.8 Statistical calculation

#### 2.8.1 Justification of sample size

The trial is designed as a single-arm, multicenter, open-label, and phase II study that aims to demonstrate therapeutic efficacy and safety of the experimental regimen pembrolizumab and trastuzumab in combination with FLOT. The co-primary end points consist of the pCR rate and the disease-free survival rate after 2 years (DFSR@2).

The efficacy assumptions can be obtained from the PETRARCA and the HER-FLOT study (14, 15). In the PETRARCA study, pCR rate in the standard FLOT arm was 12%. This was increased to 22% by adding trastuzumab to FLOT in the HER-FLOT trial and to 35% by the combination of trastuzumab, pertuzumab, and FLOT in the PETRARCA trial. We envisage that the pCR rate for pembrolizumab, trastuzumab, and FLOT will be increased to at least 30%. Hence, for the experimental regimen to be considered as a desirable candidate for further development, the pCR of 30% should be achieved. In case the pCR is 12% or less, the experimental arm would be insufficient for further development. Formally, the hypothesis testing for the first co-primary end point can be defined as H0: *P* ≤ 0.12 versus H1: *P* ≥ 0.30. The current sample size calculation is based on pCR improvement from 12% to 30% with a one-sided alpha of 5% and a beta of 20% in a Fleming single stage phase II procedure. Thus, 27 patients are required for the final analysis. Concurrently, for DFS, the respective sample size calculation would be an improvement of DFS rate at 2 years from 50% (results of the FLOT 4 trial) (31) to 70%. The hypothesis testing for the second co-primary end point can be defined as H0: *P* ≤ 0.5 versus H1: *P* ≥ 0.7. With a one-sided alpha of 0.1 and 80% power in a Fleming single stage phase II procedure, 27 patients are needed. Thus, a sample size of 27 patients is sufficient for both co-primary end points considering that the co-primary end points are homogeneous; no inflation of type II error is expected. We, therefore, keep the statistical power at 80% for both co-primary end points. Considering a dropout rate of 10%, a total number of 30 patients will be enrolled.

#### 2.8.2 Statistical analysis plan

A statistical analysis plan (SAP) will be drafted to provide details of the methods of analysis to address all study objectives. The SAP may be amended during the course of the study but will be finalized before the cutoff date for any analysis. Due to the explorative nature of this trial and the small number of patients that will be included, a descriptive statistics will be performed only (e.g., describing the distribution of the baseline demographic data with predefined subgroups).

#### 2.8.3 Analysis

Statistical analysis is based on the International Conference on Harmonization (ICH) Guidelines “Structure and Content of Clinical Study Reports” and “Statistical Principles for Clinical Trials”. Due to the explorative nature of this trial and the small number of patients, only descriptive statistics will be performed (e.g., describing the distribution of the baseline demographic data with predefined subgroups). Missing data will not be extrapolated. The number of missing values will be computed. For the time-to-event variables time to progression, PFS/DFS and OS, the Kaplan–Meier method will be used. A detailed methodology for the statistical analysis will be described in the SAP, which will be finalized before database lock. There is no full interim analysis planned for this study, due to the small sample size and the relatively short recruitment period. However, single objectives may be analyzed as soon as sufficient events are available for analysis as detailed in the SAP.

#### 2.8.4 Population for analysis

All patients receiving at least one dose of study treatment will be evaluable for safety and included in the safety population. The full analysis set (FAS) will consist of all patients that received at least one treatment dose. All efficacy analyses will be based on the FAS. Toxicity analyses will be based on the safety population.

#### 2.8.5 Primary end point

The co-primary end points are the DFSR@2 and the pCR rate. We hypothesize that the DFSR@2 will be 70% or more and as an interim efficacy analysis (to be read out after surgery of the last patient) pCR rate is 30% or greater. DFSR@2 is defined as the proportion of patients being disease free and alive 2 years after study enrollment. The pCR rate defined as the absence of residual tumor based on the pathological evaluation of the resected esophagogastric specimen in the primary by local pathology.

#### 2.8.6 Secondary end points

- Assessment of overall response rate defined as percentage of patients with CR and PR according to RECIST v1.1.
- R0 resection rate, where R0 resection is defined as a microscopically margin negative resection with no gross or microscopic tumor remains in the areas of the primary tumor and/or sampled regional lymph nodes based on evaluation by the local pathologist.
- OS is defined as time from enrollment to the date of death of any cause. If no event is observed (e.g., lost to follow-up), OS is censored at the date of last subject contact. Subjects who are alive will be censored at the last known alive dates.
- Pathological complete and subtotal regression (TRG1a/b by Becker). TRG1a/b is defined as <10% residual tumor per tumor bed based on evaluation of the resected esophagogastric specimen in the primary by local pathology.
- Perioperative morbidity and mortality.
- Safety and toxicity: AEs will be recorded and graded according to version 5.0 of National Cancer Institute Common Toxicity Criteria (NCI-CTC). Occurrence of any AE and occurrence of any serious AE (anytime during the study) will be presented. These events will also be described by nature (Primary System Organ Class and Preferred Term), severity, and causal relationship to drug administration.

#### 2.8.7 Exploratory end points

Assessment whether clinical efficacy correlates with molecularly defined subgroups (PD-L1 expression, MSI subtypes, and translational biomarkers such as immune repertoire changes or microbiota signatures).

## 3 Discussion

Considering the increasing prevalence of EGA and the limited median survival of roughly 4–5 years with current treatment standards for localized EGA, new therapeutic strategies are warranted. The addition of PD-1 inhibitors or trastuzumab to first-line chemotherapy improved ORRs in several studies (13, 24, 32). The combination of chemotherapy, trastuzumab, and pembrolizumab demonstrated an ORR of 74.4% (24, 33). Furthermore, the combination of perioperative FLOT with trastuzumab with or without pertuzumab significantly increased the pCR rate from about 15% with FLOT only to 22% or even 35% with double HER2 inhibition (AIO HER-FLOT and PETRARCA) (14, 15). Thus, the investigation of the combination of FLOT, trastuzumab, and pembrolizumab appears reasonable in the perioperative setting of HER2-positive EGA and will therefore be evaluated within the PHERFLOT trial.

The experimental regimen evaluated within the underlying clinical trial consists of the first-line standard drug combination of FLOT with the HER2-antibody trastuzumab and the PD-1 antibody pembrolizumab. Based on the available data on chemotherapy in combination with PD-L1 antibodies and HER/EGFR antibodies with PD-L1 antibodies that demonstrated the feasibility and general tolerability of the combination, this phase II trial will start with a full dose of trastuzumab, pembrolizumab, and FLOT. AEs have been broadly consistent across tumor types following monotherapy and have not demonstrated clear dose-response or exposure-response relationships. Considering that all patients will receive the standard of care chemotherapy in addition to trastuzumab and pembrolizumab, none of the patients will be withheld any standard treatment; however, AEs might limit treatment completion and may account for potential harm. To carefully evaluate potential critical toxicities, patients will be closely monitored including assessments for risk of interstitial lung disease and a continuous safety analysis for the first six recruited patients. Regarding the potential AEs and the limited benefit of immunotherapy for some patients, predictive markers to tailor treatment are urgently warranted either at baseline or early during treatment. PD-1 may serve as such biomarker in some tumor subtypes (34). In EGA, several studies reported a favorable response in PD-1 expressing subsets. Also, in terms of HER2-targeting by trastuzumab, a molecular characterization is needed since several mechanisms of treatment-induced resistance might be present upfront or will eventually develop during treatment, particularly the loss of HER2 amplification (27, 35). The recently published study results on HER2-targeting in combination with immunotherapy demonstrated an anticipated benefit for patients expressing HER2 or expressing both PD-1 and HER2 (36). Here, we will assess baseline FFPE and ctDNA for HER2, HER signaling alterations (amplifications and/or mutations in, e.g., EGFR, HER2, HER3, and PIK3CA), CTCs for HER2 and PD-L1 expression and baseline FFPE for PD-L1, MSI, and EBV to validate baseline markers with potential or likely predictive value for checkpoint-inhibition and HER2-targeting, although the coincidence of at least MSI and EBV with HER2-amplification is rare (37). Immunoprofiling by liquid biopsies will be performed prior to treatment initiation and prior to the second pembrolizumab dose to determine response predictive immune signatures, since diversification patterns can be exploited to separate responder and non-responder patients in other tumor subtypes, such as melanoma (38, 39). In addition, the intratumoral and gut microbiota was recently reported to induce response to chemo- and immunotherapies (28–30). Therefore, microbiota signatures will be analyzed and correlated with response to treatment and AEs. Overall, the translational analysis might help to determine subgroups of patients with best responses to the experimental treatment and may serve for future patient selection.

The co-primary end points are the pCR rate and the DFSR@2. The pathological response will substitute radiologically evaluated early end points, such as tumor shrinkage, that might only be evaluated with difficulty due to this localized treatment setting. Furthermore, the 2-year DFS is highly meaningful for patients undergoing surgical resection.

In summary, the PHERFLOT trial may provide evidence for a new perioperative regimen candidate in localized EGA with increased efficacy and acceptable tolerability serving as a starting point for further investigation of this innovative regimen compared to the current standard regimen FLOT within a randomized clinical phase 3 trial. The analysis of immune profiles, microbiota profiles, and expression data may contribute to the identification of urgently needed biomarkers to tailor immunotherapy in this treatment setting of EGA.

## Data availability statement

The original contributions presented in the study are included in the article/supplementary material. Further inquiries can be directed to the corresponding author.

## Ethics statement

The responsible investigator will ensure that this study is conducted in agreement with either the Declaration of Helsinki (in its current version) or the laws and regulations in its current version. The protocol has been written, and the study will be conducted according to the ICH Harmonized Tripartite Guideline for Good Clinical Practice (reference: http://www.ifpma.org/pdfifpma/e6.pdf). The protocol (AIO STO 0321) is approved by the independent ethics committee of the medical council Hamburg. Before a subject’s participation in the clinical study, the investigator must obtain written informed consent from the subject. All subjects will be informed of the aims of the study, the possible adverse events, the anticipated benefits, the procedures and possible hazards to which he/she will be exposed, and the mechanism of treatment allocation the subjects also will be informed about alternative treatments. Subjects will be informed of their insurance protection and the obligations which are linked to insurance.

## Author contributions

JT: Conceptualization, Data curation, Investigation, Writing – original draft, Writing – review & editing, Formal Analysis, Methodology. AS: Conceptualization, Data curation, Funding acquisition, Investigation, Visualization, Writing – review & editing, Formal Analysis, Resources. SA-B: Conceptualization, Resources, Writing – review & editing, Project administration. TE: Conceptualization, Resources, Writing – review & editing. TG: Conceptualization, Project administration, Resources, Writing – review & editing. BG: Conceptualization, Resources, Writing – review & editing. GH: Conceptualization, Resources, Writing – review & editing. VH: Resources, Writing – review & editing. RH: Resources, Writing – review & editing. NH: Resources, Writing – review & editing. TB: Resources, Writing – review & editing. MS: Resources, Writing – review & editing. AK: Resources, Writing – review & editing. SL: Conceptualization, Resources, Writing – review & editing. NM: Resources, Writing – review & editing. CM: Resources, Writing – review & editing. MS: Resources, Writing – review & editing. GS: Resources, Writing – review & editing. MB: Conceptualization, Data curation, Formal Analysis, Funding acquisition, Investigation, Methodology, Writing – review & editing. EG: Conceptualization, Data curation, Formal Analysis, Funding acquisition, Investigation, Project administration, Resources, Writing – review & editing.

## Glossary

**Table:**

5-FU	5-fluouracil
ALC	Absolute lymphocyte count
ANC	Absolute neutrophil count
aPTT	Activated partial thromboplastin time
AEs	Adverse events
AML	Acute myeloid leukemia
ALT	Alanine aminotransferase
ADCC	Antibody dependent cytotoxicity
AST	Aspartate aminotransferase
CRP	C-reactive protein
CT	Computed tomography
ECOG	Eastern Cooperative Oncology Group
ECG	Electrocardiogram
HER2	Epidermal receptor type 2
EBV	Epstein-Barr virus positive
EOT	End of treatment
EGA	Esophagogastric adenocarcinoma
ECIs	Events of Clinical Interest
FFPE	Formalin-fixed paraffin-embedded
FLOT	5-FU, Oxaliplatin and Taxol
GEJ	Gastroesophageal junction
HR	Hazard ratio
HP	*Helicobacter pylori*
HIV	Human immunodeficiency virus
HRCT	High resolution computed tomography
IHC	Immunohistochemistry
IGH	Immunoglobulin heavy locus
INR	International normalized ratio
ICH	International conference on harmonization
LDH	Lactate dehydrogenase
MSI	Microsatellite instable
MDS	Myelodysplastic syndrome
MRI	Magnetic resonance imaging
NCI-CTC	National Cancer Institute Common Toxicity Criteria
NGS	Next-generation sequencing
pRBC	Red blood cell
RR	Response rate
OS	Overall survival
PFS	Progression-free survival
PD	Progressive disease
PT	Prothrombin time
pCR	Pathological complete regression
SAEs	Serious adverse events
TCRβ	T-cell receptor beta
TiL	Tumor-infiltrating lymphocytes
WBC	White blood cell
WOCBP	Women of childbearing potential
